# Comparison of lung cancer cell lines representing four histopathological subtypes with gene expression profiling using quantitative real-time PCR

**DOI:** 10.1186/1475-2867-10-2

**Published:** 2010-01-21

**Authors:** Takashi Watanabe, Tomohiro Miura, Yusuke Degawa, Yuna Fujita, Masaaki Inoue, Makoto Kawaguchi, Chie Furihata

**Affiliations:** 1 Department of Chemistry and Biological Science, School of Science and Engineering, Aoyama Gakuin University, Kanagawa 229-8558, Japan; 2 Department of Chest Surgery, Niigata Rosai Hospital, Japan Labor Health and Welfare Organization, Niigata 942-8502, Japan; 3 Department of Pathology, Niigata Rosai Hospital, Japan Labor Health and Welfare Organization, Niigata 942-8502, Japan

## Abstract

**Background:**

Lung cancers are the most common type of human malignancy and are intractable. Lung cancers are generally classified into four histopathological subtypes: adenocarcinoma (AD), squamous cell carcinoma (SQ), large cell carcinoma (LC), and small cell carcinoma (SC). Molecular biological characterization of these subtypes has been performed mainly using DNA microarrays. In this study, we compared the gene expression profiles of these four subtypes using twelve human lung cancer cell lines and the more reliable quantitative real-time PCR (qPCR).

**Results:**

We selected 100 genes from public DNA microarray data and examined them by DNA microarray analysis in eight test cell lines (A549, ABC-1, EBC-1, LK-2, LU65, LU99, STC 1, RERF-LC-MA) and a normal control lung cell line (MRC-9). From this, we extracted 19 candidate genes. We quantified the expression of the 19 genes and a housekeeping gene, *GAPDH*, with qPCR, using the same eight cell lines plus four additional validation lung cancer cell lines (RERF-LC-MS, LC-1/sq, 86-2, and MS-1-L). Finally, we characterized the four subtypes of lung cancer cell lines using principal component analysis (PCA) of gene expression profiling for 12 of the 19 genes (*AMY2A*, *CDH1*, *FOXG1*, *IGSF3*, *ISL1*, *MALL*, *PLAU*, *RAB25*, *S100P*, *SLCO4A1*, *STMN1*, and *TGM2*). The combined PCA and gene pathway analyses suggested that these genes were related to cell adhesion, growth, and invasion. *S100P *in AD cells and *CDH1 *in AD and SQ cells were identified as candidate markers of these lung cancer subtypes based on their upregulation and the results of PCA analysis. Immunohistochemistry for S100P and RAB25 was closely correlated to gene expression.

**Conclusions:**

These results show that the four subtypes, represented by 12 lung cancer cell lines, were well characterized using qPCR and PCA for the 12 genes examined. Certain genes, in particular *S100P *and *CDH1*, may be especially important for distinguishing the different subtypes. Our results confirm that qPCR and PCA analysis provide a useful tool for characterizing cancer cell subtypes, and we discuss the possible clinical applications of this approach.

## Background

Lung cancer is the leading cause of cancer-related death in men and women worldwide and continues to increase in frequency. Currently, a diagnosis of lung cancer is generally based on histopathological findings. Lung cancers are generally classified as either small-cell lung carcinoma (SC) or non-small-cell lung carcinoma (NSCLC). NSCLC is further classified into three histopathological subtypes: adenocarcinoma (AD), squamous cell carcinoma (SQ), and large cell carcinoma (LC). However, progression, metastatic susceptibility, therapeutic and radiation therapy sensitivity, and prognosis cannot be fully predicted based on initial histopathological observations. Molecular characterization of tumors, by assaying gene expression using techniques such as DNA microarray analysis, has the potential to significantly inform medical care that is otherwise based on surgical pathology and oncology. Using this technology, it may be possible to identify clinically important subsets of tumors that would otherwise be indistinguishable by conventional histopathological assessment. In principle, expression profiling should identify tumors that are more likely to invade, relapse, and metastasize, and the approach should allow improved prediction of responses to specific therapeutic regimens and clinical outcomes [1-3]. However, recent publications have raised concerns about the reliability of microarray technology for analyzing differential expression, because of the lack of reproducibility across laboratories and platforms despite the use of highly similar protocols [4]. Initial investigations (e.g., 2000-2003) highlighted discrepancies in gene expression analyzed with different microarray technologies [5]. Although a considerable number of studies have used DNA microarrays to genetically identify lung cancer patients and lung cancer cells [1-3,6-10], marker gene candidates have varied depending on the report.

Quantitative real-time PCR (qPCR) is generally considered the "gold-standard" assay for measuring gene expression and is often used to confirm microarray data [11]. qPCR is the most sensitive technique for detection and quantification of mRNA targets [12]. Recently, it has been suggested that qPCR may be a simpler, more reliable, and more reproducible method than DNA microarrays [13]. qPCR has been used as a supplementary technique for characterizing lung cancer cells [14]. The recent development of DNA databases and bioinformatics techniques has made it possible to determine gene pathways and gene networks [15]. Statistical analyses, such as principal component analysis (PCA), have recently proven useful in this field. Establishing molecular profiles of the four histopathological subtypes of lung cancer cells in relation to gene networks and statistical analysis would be a valuable and meaningful undertaking. Because the analysis of DNA microarrays is expensive and complex, it is often not practical for routine diagnosis to use high-throughput DNA microarrays containing more than 10,000 genes. A diagnostic approach designed for less than 100 marker genes using either a smaller, less-expensive DNA microarray or qPCR would be more practical. To classify the four histopathological subtypes, we selected 100 candidate marker genes that showed relatively consistent differential expression in reports that analyzed a total of 580 clinical lung cancer tissues and 64 lung cancer cell lines [1-3,6-10]. We first selected candidate genes using DNA microarrays and then quantified their expression by qPCR. Although clinical application is the ultimate goal, there are some issues to consider when examining clinical tissues with DNA microarrays or qPCR. First, tissues contain varying amounts of contamination from neighboring stromal cells. Second, RNA amplification is required if the amount of clinical tissue is limited, for instance when samples are obtained by microdissection of cancer cells. While these issues are not problematic for analyzing lung cancer cell lines, they become significant barriers when analyzing clinical samples. Finally, the use of epithelial tissue from sites adjacent to tumors as the normal control has drawn criticism [16], as this tissue often includes histologically normal but genetically abnormal cells [17].

In this study, we first selected 100 genes from published studies and used DNA microarrays to examine their expression in eight test cell lines (A549 [AD], ABC-1 [AD], EBC-1 [SQ], LK-2 [SQ], LU65 [LC], LU99 [LC], STC 1 [SC], RERF-LC-MA [SC]) representing four histopathological subtypes of lung cancer cells plus a normal control lung cell line (MRC-9). From this, we identified 19 candidate genes for subtype-specific markers. Second, we quantified the expression of these 19 genes in the different cell lines using qPCR. Third, we evaluated the 19 genes with an additional four validation lung cancer cell lines (RERF-LC-MS [AD], LC-1/sq [SQ], 86-2 [LC], and MS-1-L [SC]) and MRC-9 cell by qPCR. Fourth, we analyzed the data using statistical, bioinformatics, PCA, and gene pathway analysis (Ingenuity Pathways Analysis, IPA). We selected 12 optimal marker genes and demonstrated that these profiles could discriminate the four histopathological subtypes of tumors. In addition, we confirmed the results using immunohistochemical analysis.

## Results

### Identification of candidate genes by microarray analysis

We selected 100 genes from public DNA microarray data [1-3,6-10] and examined their expression in eight lung cancer cell lines (A549 [AD], ABC-1 [AD], EBC-1 [SQ], LK-2 [SQ], LU65 [LC], LU99 [LC], STC 1 [SC], RERF-LC-MA [SC]) and a normal control (MRC-9) by DNA microarray analysis. After eliminating low-expressing genes, we calculated the expression ratio of each gene in each cancer cell line relative to the normal cell line. From this, we identified 18 differentially expressed candidate genes based on the results of a Dunnett's test (Table 1). Another one gene, ISL1, was added as a tentative candidate gene because it had more than 10-fold-higher expression in one cell line than any other line (Table 1). The microarray results were deposited in the CIBEX microarray database (accession CBX 100).

**Table 1:** Selection of candidate genes by DNA microarray

Gene	Cell lines by histopathological subtype							
	
	AD		SQ		LC		SC	
	
	A549	ABC-1	EBC-1	LK-2	LU65	LU99	STC 1	RERF-LC-MA
**AMY2A**	-1.22	0.87	3.68 **	0.68	2.50 *	1.90	2.91 *	1.36

**BEX1**	3.25	5.69	7.36 **	3.08	-0.17	2.86	4.72 *	5.72

**CDH1**	1.85	3.70 **	1.87	3.85 **	-0.41	5.20	0.48	-0.70

**CSTA**	2.54 **	4.79 **	-1.06	0.80	0.09	-1.93	2.18 **	0.33

**DUSP4**	4.24 **	2.34 **	4.91 **	1.26	4.12 **	0.96	-3.21	2.16 *

**FOSL1**	-1.26	-1.73	3.27 **	-2.62	2.10 **	2.44 **	-4.13	2.17 **

**FOXG1**	-0.19	3.78	-0.20	0.65	-0.16	1.14	5.63 **	0.17

**HMGA1**	-0.59	0.24	2.82 **	0.82	1.68 **	3.24 **	0.49	2.81 **

**IGSF3**	5.61 **	4.86	-0.12	6.35 *	0.19	1.28	4.71	4.62

**INADL**	2.44	3.70	2.50	7.65 **	0.48	1.48	0.80	2.30

**ISL1**	0.31	-0.45	0.08	2.21	4.61	0.10	-0.74	3.96

**MALL**	2.20 **	2.39 **	2.44 **	-0.07	3.86 **	1.54 *	N.D.	2.36 **

**PLAU**	-3.61 **	-7.21 **	-0.02	-6.75 **	-1.82 **	-1.17 **	-6.95 **	-1.62 **

**RAB25**	-0.39	6.39 **	3.37	5.33 **	0.65	0.44	-2.87	-1.18

**S100A2**	-0.70	-0.02	4.73 **	0.08	2.95 **	1.66	-1.01	4.76 **

**S100P**	6.28 **	8.57 **	-0.47	5.00	6.80 **	0.38	0.39	0.13

**SLCO4A1**	0.13	2.92	2.81	1.52	4.74 *	6.67 **	2.83	1.78

**STMN1**	-1.44	1.41 **	1.49 **	1.41 **	0.44	0.68	2.07 **	2.21 **

**TGM2**	-0.56	-2.93 **	0.46	-5.61 **	-0.39	-1.99	-7.62 **	-5.52 **

Total RNA was extracted from each cultured cell line and reverse-transcribed to produce cDNA. cDNA samples from cancer cells (labeled with Alexa 555) and from normal cells (labeled with Alexa 647) were mixed and hybridized to a DNA microarray that was then scanned with a DNA microarray scanner (n = 6). The ratio of cancer cells to control cells, based on the relative intensities of the two fluorescence signals, was calculated using ArrayGauge. *p < 0.05, **p < 0.01 with Dunnett's test.

### Quantification of 19 candidate genes by qPCR

**Figure 1:**
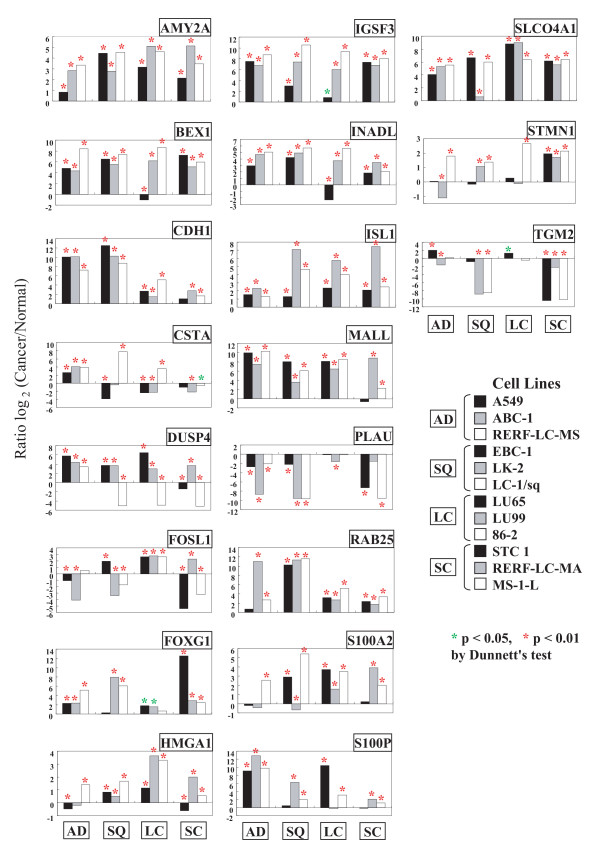
**Quantification and validation of 19 genes by qPCR**. The expression of 19 genes in 8 test lung cancer cell lines (black and gray) and four validation cell lines (white) was quantified by qPCR and compared to a normal control cell line (MRC-9).

Using qPCR, we quantified the expression of the 19 candidate genes in the same eight test cell lines (A549, ABC-1, EBC-1, LK-2, LU65, LU99, STC 1, RERF-LC-MA) and the normal cell line (MRC-9) (Figure. 1). This gave a total of 152 data points (19 × 8) from each of the DNA microarray and qPCR analyses. Increases or decreases in 118 data points from qPCR were consistent with those from the DNA microarray analysis. Furthermore, qPCR provided more sensitive data than the DNA microarray analysis (Table 1 and Figure. 1). Using qPCR, two AD cell lines showed consistent and significant upregulation in 12 genes (*AMY2A*, *BEX1*, *CDH1*, *CSTA*, *DUSP4*, *FOXG1*, *IGSF3*, *INADL*, *ISL1*, *MALL*, *S100P*, and *SLCO4A1*) and downregulation in *PLAU*. Two SQ cell lines showed upregulation in 11 genes (*AMY2A*, *BEX1*, *CDH1*, *DUSP4*, *HMGA1*, *IGSF3*, *INADL*, *ISL1*, *MALL*, *RAB25, *and *SLCO4A1*) and downregulation in *PLAU*. Two LC cell lines showed upregulation in 12 genes (*AMY2A*, *CDH1*, *DUSP4*, *FOSL1*, *FOXG1*, *HMGA1*, *IGSF3*, *ISL1*, *MALL*, *RAB25*, *S100A2*, and *SLCO4A1*) and downregulated in *CSTA*. Two SC cell lines showed upregulation in nine genes (*AMY2A*, *BEX1*, *FOXG1*, *IGSF3*, *INADL*, *ISL1, RAB25*, *SLCO4A1*, and *STMN1*) and downregulation in *TGM2 *(Figure 1).

### Evaluation by qPCR using validation cell lines

We evaluated the expression profiling of the 19 genes using four validation cell lines (RERF-LC-MS [AD], LC-1/sq [SQ], 86-2 [LC], and MS-1-L [SC]) and the normal control (MRC-9). The results of expression profiling are shown in Figure 1. The validation AD cell line showed similar upregulation in the same 12 genes (*AMY2A*, *BEX1*, *CDH1*, *CSTA*, *DUSP4*, *FOXG1*, *IGSF3*, *INADL*, *ISL1*, *MALL*, *S100P*, and *SLCO4A1*) and downregulation in *PLAU*. The validation SQ cell line showed similar upregulation in 10 genes (*AMY2A*, *BEX1*, *CDH1*, *HMGA1*, *IGSF3*, *INADL*, *ISL1*, *MALL*, *RAB25 *and *SLCO4A1*) and downregulation in *PLAU*. The validation LC cell line showed similar upregulation in 10 genes (*AMY2A*, *CDH1*, *FOSL1*, *HMGA1*, *IGSF3*, *ISL1*, *MALL*, *RAB25*, *S100A2*, and *SLCO4A1*). The validation SC cell line showed similar upregulation in the same nine genes (*AMY2A*, *BEX1*, *FOXG1*, *IGSF3*, *INADL*, *ISL1, RAB25*, *SLCO4A1*, and *STMN1*) and downregulation in *TGM2*. Thus, the concordance rates were 100% for the AD, and SC validation lines, 92% (11/12) for the SQ line, 77% (10/13) for the LC line, and 92% (44/48) overall. *CSTA*, *DUSP4*, and *S100P *were upregulated consistently in only AD cells, and *FOSL1 *and *S100A2 *were upregulated in only LC cells. *STMN1 *was upregulated and *TGM2 *was downregulated in only SC cells.

### Principal component analysis (PCA)

**Figure 2:**
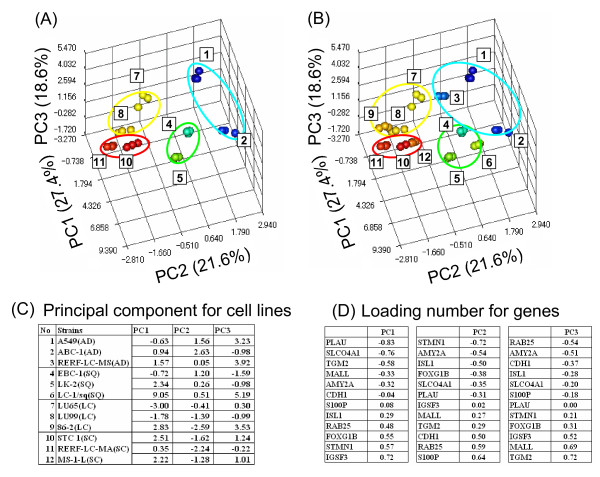
**Principal component analysis of cell lines with 12 genes based on qPCR data**. PCA differentiates four histopathological subtypes by three-dimensional expression clustering. The values of triplicate qPCR assays for each sample were analyzed. Results of PCA are shown in the three-dimensional contribution scores for component numbers 1, 2, and 3 (PC1, PC2, and PC3), which discriminate the four histopathological clusters. Data are shown for the eight test cell lines alone (A) and in combination with the four validation cell lines (B). The contribution scores were produced by conversion from each eigenvector value.

To classify the four histopathological subtypes by PCA we tried and selected various set of qPCR results from 19 genes. The four histopathological subtypes were optimally classified by PCA using 12 genes (*AMY2A*, *CDH1*, *FOXG1*, *IGSF3*, *ISL1*, *MALL*, *PLAU*, *RAB25*, *S100P*, *SLCO4A1*, *STMN1*, and *TGM2*) in the eight test cell lines, with the loading number of components 1, 2, and 3 (PC1, PC2, and PC3; 3 dimensions) shown in Figure 2(A). Using the same frame, the four subtypes were also classified using all 12 test and validation cell lines, as shown in Figure 2(B). Figure 2(B) shows that the four subtypes were divided into two prominent groups corresponding to positive PC2 values (AD and SQ) and negative PC2 values (LC and SC). In Figure 2(C), which shows the principal component for the cell lines (PC1, PC2, and PC3), two of the three AD cells lines were close, whereas the third cell line was close in PC2 to the other two cell lines, but separate in PC1 and PC3. Specifically, A549 and RERF-LC-MS cells were close in PC1, PC2, and PC3, whereas ABC-1 cells showed a distinct, small negative value in PC3. Two SQ cell lines, EBC-1 and LK-2, were close in PC1, PC2, and PC3, whereas one SQ cell line, LC-1/sq, showed a distinct, large positive value in PC1 and PC3. Two close AD cell lines and two close SQ cell lines were different in PC3 with a positive value in AD and a negative value in SQ. ABC-1 (AD) and LC-1/sq (SQ) were also separated in different directions. Two LC cell lines, LU65 and LU99, were close in PC1, PC2, and PC3, whereas one LC cell line, 86-2, showed intermediate separation in PC1 and PC3. The three SC cell lines were close to each other. Two close LC cell lines and three SC cell lines were different in PC1, with a negative value for LC and a positive value for SC. The 86-2 cell line (LC) was different in PC3 from the SC cell lines. Subtypes were characterized by PC1-3 and loading number. In Figure 2(D), which shows the loading number for genes, PC1 was negatively correlated with the expression of *PLAU*, *SLCO4A1*, and *TGM2*, and positively correlated with *IGSF3*, *STMN1*, *FOXG1*, and *RAB25*. PC2 was negatively correlated with the expression of *STMN1*, *AMY2A*, and *ISL1*, and positively correlated with *S100P*, *RAB25*, and *CDH1*. PC3 was negatively correlated with the expression of *RAB25 *and *AMY2A*, and positively correlated with *TGM2*, *MALL*, and *IGSF3*.

### Gene networks and gene pathways

**Figure 3:**
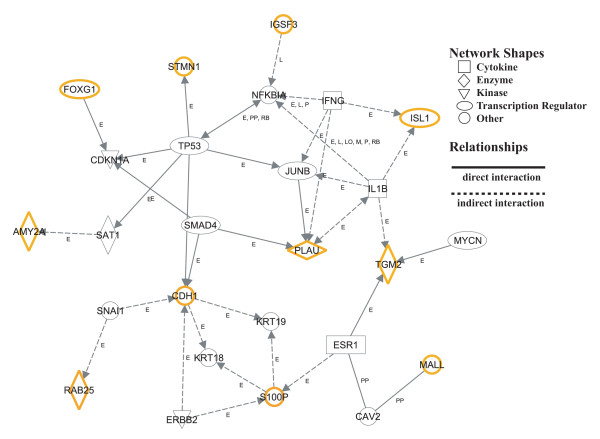
**Gene networks and pathways of 11 genes from PCA analysis**. The network was analyzed using Ingenuity Pathways Analysis software and is displayed graphically as nodes (genes/gene products) and edges (the biological relationships between the nodes). Nodes are displayed using shapes that represent the functional class of the gene product, as indicated in the key. Edges are displayed with labels that describe the nature of the relationship between the nodes (E, expression; L, proteolysis; LO, localization; M, biochemical modification; P, phosphorylation/dephosphorylation; PP, protein-protein binding; RB, regulation of binding).

To further understand the biological networks of the 19 genes, we next analyzed their biological interactions using the Ingenuity Pathways Analysis (IPA) tool. Ten networks were extracted from each cancer cell line. Table 2 shows a major network (network 1) that contained 12 of the 19 candidate genes (*AMY2A*, *BEX1*, *CDH1*, *DUSP4*, *FOSL1*, *FOXG1*, *HMGA1*, *ISL1*, *PLAU*, *S100P*, *STMN1*, and *TGM2*), including 7 of the 12 PCA genes (*CDH1*, *FOXG1*, *ISL1*, *PLAU*, *S100P*, *STMN1*, and *TGM2*), using ABC-1 cells as a representative cell line. The other 11 cell lines showed a similar network 1, with various gene-specific increases and decreases and with slightly different top functions. The other nine networks were smaller (not shown). Figure 3 shows the gene networks of 11 of the 12 PCA genes (except *SLCO4A1*) based on IPA results. Link from *SLCO4A1 *to the present gene networks was not extracted by IPA. The connection including *CDH1*, *PLAU*, and *SMAD4 *suggested to be related to cell adhesion by IPA. The connection including *TGM2*, *IL1B*, and *PLAU *and the connection including *RAB25*, *SNAI1*, and *CDH1 *were suggested to be related to tumor invasion by IPA. *STMN1 *was suggested to influence cell motility, and *S100P *was suggested to be associated with cell growth by IPA.

**Table 2:** IPA network 1 of ABC-1 cell

Cell	Genes in network	Score	Focus genes	Top functions
ABC-1	AGER, Ap1, **↑ BEX1**, CDC42EP5, **↑ CDH1**, Ck2, deoxycholate, **↑ DUSP4**, ERK, **↓ FOSL1**, **↑ FOXG1**, FSH, FXYD5, GDF15, HMGA1, Il8r, **↑ ISL1**, Jnk, LCN2, MAD2L2, Mapk, MGAT3, MKP2/5, NFkB, PDGF BB, PI3K, **↓ PLAU**, PTPRF, PVR, RAGE, S100A1, **↑ S100P**, SLC12A6, **↓ STMN1**, **↓ TGM2**	28	11	Cancer, Cellular Movement, Cellular Growth and Proliferation

Biologically relevant network 1 extracted by IPA is shown for ABC-1 cell as a representative. ↑ marks represent upregulated genes and ↓ marks downregulated genes. genes.

### Immunohistochemistry

**Figure 4:**
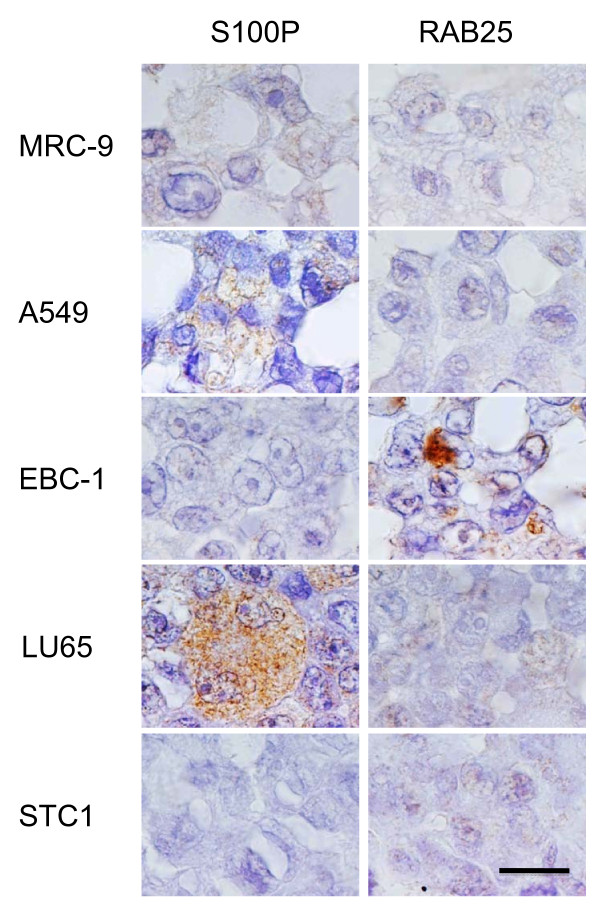
**Immunohistochemical analysis**. Representative images of immunohistochemical analysis of S100P and RAB25 protein in four lung cancer cell lines (A549 [AD], EBC-1 [SQ], LU65 [LC], and STC 1 [SC]) and the normal cell line MRC-9. Bar, 20 μm.

Routine immunohistochemical studies were performed in four test cell lines (A549, EBC-1, LU65, and STC 1) and the control (MRC-9), to define their histopathological classification (Figure 4). S100P protein was expressed in the cytoplasm of A549 and LU65 cells. RAB25 protein was expressed in the cytoplasm of EBC-1 cells. These results were consistent with the gene expression data for *S100P *and *RAB25 *(Figure 1).

## Discussion

We compared four histopathological subtypes of 12 lung cancer cell lines using a statistical processing method, PCA, which is based on gene expression profiling determined by qPCR. Four subtypes were optimally classified by PCA using 12 genes (*AMY2A*, *CDH1*, *FOXG1*, *IGSF3*, *ISL1*, *MALL*, *PLAU*, *RAB25*, *S100P*, *SLCO4A1*, *STMN1*, and *TGM2*) from the 19 candidate genes shown in Figure 1. PCA analysis revealed that the loading number of component 1 (PC1) was negatively correlated with the expression of *PLAU*, *SLCO4A1*, and *TGM2*, and positively correlated with *IGSF3*, *STMN1*, *FOXG1*, and *RAB25*. The loading number of component 2 (PC2) was negatively correlated with the expression of *STMN1*, *AMY2A*, and *ISL1*, and positively correlated with *S100P*, *RAB25*, and *CDH1*. The loading number of component 3 (PC3) was negatively correlated with the expression of *RAB25 *and *AMY2A*, and positively correlated with *TGM2*, *MALL*, and *IGSF3*. The four subtypes were divided into two prominent groups with PC2, corresponding to positive PC2 values (AD and SQ) and negative PC2 values (LC and SC). Because PC2 was positively correlated with the expression of *CDH1*, *S100P*, and *RAB25*, these genes may be significant in the classification of the four subtypes. Three SC cell lines were close to each other in PC1, PC2, and PC3. As the presence of subclasses in AD and SQ clinical tissues was suggested [6,7,18], it was probable that there was some diversity in the present AD and SQ cell lines. Gene expression of these 12 genes was generally consistent with some exceptions in the four subtypes. Even when gene expression was not fully consistent among the subtypes, PCA with the present 12 genes could be used effectively to classify the four subtypes.

Using DNA microarrays and qPCR, Kuner et al. [19] recently compared gene expression in 42 AD and 18 SQ clinical tumor samples and systematically analyzed their expression patterns using gene ontology. This group identified 14 tight junction genes and 9 epithelial-mesenchymal transition genes that were upregulated or downregulated in AD samples, SQ samples, or both. Among these genes, the epithelial-mesenchymal transition gene *CDH1, *which codes for E-cadherin, was upregulated in both AD and SQ samples. We also examined gene ontology. Although our overall results were unclear, our data suggest that *CDH1 *is associated with cell adhesion, and that the AD and SQ cell lines are associated with greater cell adhesion, while LC and SC cell lines are associated with weaker cell adhesion. Taken together, these studies demonstrate a remarkable upregulation of *CDH1 *in AD and SQ cells, but not LC and SC cells, making this a candidate marker for differentiating lung cancer subtypes. *CDH1 *was the only gene studied in both the Kuner et al. report and in ours.

Using cDNA microarrays and gene ontology, Inamura et al. [18] analyzed 48 SQ clinical tissue samples and classified them into two subclasses. Subclass A genes were related to processes such as cell proliferation and cell cycle progression, while subclass B genes were related to processes such as the MAPKKK cascade and apoptosis. They focused on 30 possible marker genes that were completely different from the 23 genes identified in the Kuner et al. report and the 12 genes we studied.

Using bioinformatics, Kim et al. [20] extracted differentially expressed lung cancer candidate genes from published data examined by SAGE method. Next, they used qPCR to compare candidate gene expression in 18 AD and 18 SQ samples from microdissected clinical tissues. They extensively examined seven genes and identified two, *CBLC *and *CYP24A1*, as novel candidate biomarkers for AD and SQ cells. They also suggested that *S100P, *which encodes S100 calcium-binding protein P, may be a good biomarker for AD cells. The expression ratio of *S100P *in cancer/normal cells was high in AD samples and low in SQ samples. In our study, all three AD cell lines showed a robust increase in *S100P *expression, while the three SQ cell lines showed less or no increase. Taking our data and the Kim et al. data together, the remarkable and specific upregulation of *S100P *in AD cells suggests that this is a candidate marker for distinguishing the AD subtype. Although the Kim and Kuner groups both analyzed AD and SQ samples, their gene sets (7 and 30, respectively) were non-overlapping.

Identification of molecular markers often leads to important clinical applications, such as earlier diagnosis, better prognosis, and more effective drug targeting. Although numerous papers examining lung cancer tissues and/or lung cancer cell lines using DNA microarrays and/or qPCR have been published e.g.[1-3,6-10,19,20], lung cancers still lack reliable molecular markers [20]. The genes examined varied between paper, and the results were not necessarily consistent. This variability may result from technical limitations, differences in methodology, and the broad biological heterogeneity of lung cancers themselves. Continued accumulation of data will help resolve this question. The studies described were conducted primarily with AD and SQ samples. Many fewer studies looked at LC and SC samples, and direct comparison of all four histopathological subtypes using the same method(s) was rare. Our study is unique because we examined 12 lung cancer cell lines representing all four subtypes, and we used both qPCR and PCA of 12 genes (*AMY2A*, *CDH1*, *FOXG1*, *IGSF3*, *ISL1*, *MALL*, *PLAU*, *RAB25*, *S100P*, *SLCO4A1*, *STMN1*, and *TGM2*). Although none of these 12 genes represent novel candidate markers because they were all selected from earlier microarray studies [1-3,6-10], this is the first report that systematically analyzed them together in all four subtypes.

The gene network was analyzed using Ingenuity Pathways Analysis software and is displayed graphically in Figure 3. The first connection including *CDH1*, *PLAU*, and *SMAD4 *was suggested to be related to cell adhesion [21,22]. It was reported that *SMAD4 *reduced the expression level of endogenous *PLAU *[21] and induced *CDH1 *expression [22]. The second connection including *RAB25*, *SNAI1*, and *CDH1 *was suggested to be related to tumor invasion. It was reported that *RAB25 *enhanced the ability of tumor cells to invade the extracellular matrix [23]. The first and second connection may be applicable to AD and SQ cell lines in this study. It was reported that *STMN1 *influenced cell motility [24] and *S100P *was associated with cell growth [25]. *STMN1 *and *S100P *may work in SC cell lines and AD cell lines, respectively, in this study. The third connection including *TGM2*, *IL1B*, and *PLAU *was suggested to be tumor invasion. It was reported that *IL1B *increased the expression level of *TGM2 *[26], which might be involved in establishing a barrier to tumor spreading [27]. The third connection may not be effective in cell lines in this study, because *TGM2 *was rather downregulated in this study.

Six of the genes analyzed (*CDH1*, *PLAU*, *RAB25*, *S100P*, *STMN1*, and *TGM2*) have attracted recent attention relating to therapeutic drug sensitivity and prognosis. In gene expression profiling studies of lung cancer cell lines to study therapeutic drug sensitivity, *PLAU *and *CDH1 *have been suggested as novel biomarkers of cetuximab sensitivity [28], and *TGM2 *was suggested as a potential marker of doxorubicin sensitivity [27]. *STIMN1 *was reported to be a novel therapeutic target for anticancer activity [29]. Additionally, *RAB25 *may be linked to tumor aggressiveness and metastasis [23], and *S100P *may be a diagnostic marker of non-small-cell lung cancer [30,31]. *PLAU *has also been examined in relation to lung cancer prognosis [32]. The set of 12 well-characterized cell lines described in this study, representing the four histopathological subtypes, should prove useful for screening therapeutic drugs and their effects on specific genes.

We performed additional immunohistochemical studies to examine S100P and RAB25 (Figure 4). The results were generally consistent with the gene expression data (Figure 1). In immunostained tumor tissues, AD cells showed immunostaining of S100P in the cytoplasm and the nucleus, while SQ cells showed immunostaining of RAB25 in the cytoplasm. The localization of S100P and RAB25 in tumor tissues was similar to that in cultured cells (data not shown).

Although DNA microarray technology is a powerful tool for characterizing gene expression on a genome scale, issues of reliability, reproducibility, and the correlation of data across different DNA microarrays still need to be addressed. Recently, qPCR was described as being simpler and more reliable than DNA microarrays [13]. Our experiments confirmed that qPCR was simpler, more reproducible, and more reliable than DNA microarrays. In the future, identification of reliable marker genes will hopefully allow for the development of automatic qPCR systems for routine clinical cancer diagnosis.

We examined the characteristics of four histopathological subtypes in lung cancer cell lines using both statistical analysis and biological network analysis. In the future, studies with cultured lung cancer cells should improve our ability to predict the response of different lung cancer types to specific therapeutic regimens.

## Conclusions

Our results showed that the four histopathological subtypes, represented by 12 lung cancer cell lines, were well characterized by qPCR and PCA using 12 genes: *AMY2A*, *CDH1*, *FOXG1*, *IGSF3*, *ISL1*, *MALL*, *PLAU*, *RAB25*, *S100P*, *SLCO4A1*, *STMN1*, and *TGM2*. Based on their upregulation and the results of the PCA analysis, *S100P *and *CDH1 *were identified as candidate markers for AD tumors and for AD and SQ tumors, respectively.

## Methods

### Cell lines and RNA isolation

The human lung cancer cell lines ABC-1 (AD), RERF-LC-MS (AD), EBC-1 (SQ), LK-2 (SQ), LC-1/sq (SQ), LU65 (LC), LU99 (LC), STC 1 (SC), RERF-LC-MA (SC), MS-1-L (SC), and MRC-9 (normal control lung cell line) were purchased from the Japanese Collection Research Resources Bank (JCRB, Osaka Japan). The 86-2 (LC) lung cancer cell line was purchased from Riken Bioresource Center (Tsukuba, Japan), and the A549 (AD) lung cancer cell line was a generous gift provided by Dr. Akira Yasui of Tohoku University (Sendai, Japan). Total RNA samples were isolated from each cultured cell line using Micro Smash MS-100 (Tomy Digital Biology Co., Ltd. Tokyo) and QuickGene-800 (Fujifilm, Tokyo). RNA quality assurance was performed by measuring the 260:280 nm ratio with a spectrophotometer (NanoDrop Technologies, LLC, Wilmington, DE, USA) and by gel electrophoresis using the Bioanalyzer and Agilent RNA 6000 Nano kit (Agilent Technologies Inc., Santa Clara, CA, USA).

### DNA microarray design and production

The 100 candidate marker genes, which were selected based on previous reports [1-3,6-10], are shown in additional file 1http://www.chem.aoyama.ac.jp/Chem/ChemHP/Furihatalab/. Synthesis of newly designed probes (Japan Patent No. 2007-234363) was outsourced to Invitrogen Corp. (Carlsbad, CA, USA). The probes were spotted onto a GeneSlide platform (Toyo Kohan Co., Ltd. Tokyo) using a Genex Arrayer Type-M (Kaken Geneqs, Inc., Chiba, Japan). GeneSlides were prehybridized at 80°C for 1 hour, washed in 2× SSC/0.2% SDS and then ultrapure water, and then dried by centrifugation.

### cDNA synthesis and gene expression profiling by DNA microarray

Alexa-labeled target cDNA was prepared from 20 μg total RNA using a SuperScript Plus Indirect cDNA System kit (Invitrogen Corp., Carlsbad, CA, USA). cDNA obtained from cancer cell lines was labeled with Alexa 555, and cDNA obtained from the control cell line was labeled with Alexa 647. The two Alexa-labeled cDNA samples were mixed and hybridized to a single DNA microarray that was then scanned in a DNA microarray scanner (FLA-8000, Fujifilm). To identify upregulated and downregulated genes, the ratio of relative intensities of the two fluorophores (Alexa 555: Alexa 647) was calculated after global normalization using ArrayGauge (Fujifilm). DNA microarray array data were deposited into the Center for Information Biology Gene Expression Database (CIBEX; accession: CBX 100).

### Quantification of genes using qPCR

**Figure 5:**
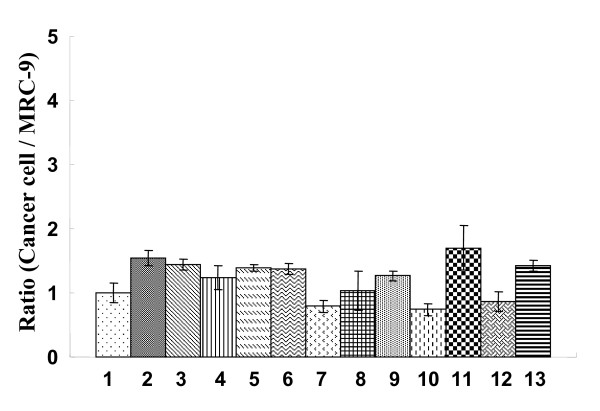
**Relative expression of *GAPDH***. Total RNA was extracted from each of the 12 lung cancer cell lines and reverse-transcribed to produce cDNA. *GAPDH *expression was determined by qPCR in triplicate assays. Results are shown as the mean ± S.D. Numbers indicate cell lines, 1: MRC-9 [normal control], 2: A549 [AD], 3: ABC-1 [AD], 4: RERF-LC-MS [AD], 5: EBC-1 [SQ], 6: LK-2 [SQ], 7: LC-1-sq [SQ], 8: LU65 [LC], 9: LU99 [LC], 10: 86-2 [LC], 11: STC 1 [SC], 12: RERF-LC-MA [SC] and 13: MS-1-L [SC].

cDNA was prepared from 2.5 μg total RNA using the SuperScript first-strand synthesis system from an RT-PCR kit (Invitrogen Corp., Carlsbad, CA, USA). qPCR amplifications were performed with triplicate assays using the SYBR Green I assay in an Opticon 2 thermal cycler (MJ Research, Inc., Waltham, MA, USA). The reactions were carried out in a 96-well plate in 20-μl reactions containing 2× SYBR Green Master Mix (Applied Biosystems, Lincoln Centre Drive Foster City, CA, USA), 2 pmol of each forward and reverse primer, and a cDNA template corresponding to 400 pg total RNA. The primer sequences and Ct values of the 19 candidate genes and *GAPDH *(a housekeeping gene as an internal control) are shown in Table 3. SYBR Green PCR conditions were 95°C for 10 minutes, followed by 45 cycles of 95°C for 10 seconds, 58°C for 50 seconds, and 72°C for 20 seconds. In each assay, a standard curve was calculated concurrently with the examined samples. In the preliminary experiment, the group expressing the highest amount of product was selected for each gene and used as the standard sample in the subsequent assay. Each standard curve consisted of six serial dilutions (1, 1/5, 1/25, 1/125, 1/625, and 1/3125) of the selected standard cDNA for each gene. The relative quantitative value of each sample was determined with the 1/25-diluted cDNA and was normalized to *GAPDH *as described previously [33]. Relative *GAPDH *expression in the experimental cell lines is shown in Figure 5.

**Table 3:** Primer sequences of 20 genes examined in the study

No.	Symbol	Ct	Left	Right
1	*AMY2A*	25-31	GGGTTCGTATTTATGTGGATGC	GGGTTCGTATTTATGTGGATGC
2	*BEX1*	21-31	GCCTAGAGGAAATCGTAGGCGGTTC	CTCTCATCCTTGCCTGTGGTTCTCC
3	*CDH1*	22-33	TGGGCAGCTATCCAGTGACTTGTTC	CTGTCTTTGGCTGCAGCACTTTAGG
4	*CSTA*	25-35	CACTTTGGTTCCAGCATCCTGTC	ACAATCTCCTGGATTTCTGGAGTG
5	*DUSP4*	20-29	CTGCTTCTCAGTGGCAACAAAC	CCGTAGCATGCAGATGTCAAGG
6	*FOSL1*	18-22	TAAGGCGCGAGCGGAACAAG	TCGCTGCAGCCCAGATTTCTC
7	*FOXG1*	23-36	GCGCCAGCAGCACTTTGAGTTAC	TGGTTGTTGCCCAGCGAGTTC
8	*GAPDH(GAPD)*	21-22	AGTAGAGGCAGGGATGATGTTC	CTTTGGTATCGTGGAAGGACTC
9	*HMGA1*	17-20	GCGAAGTGCCAACACCTAAGAGACC	CCTTGGTTTCCTTCCTGGAGTTGTG
10	*IGSF3*	24-35	GACCTCATTGCGCATTGTCTAC	CATGTCCTAGAATGCGCCTAG
11	*INADL*	23-31	AGAATGGACTTGGACTCAGCCTTGC	CATCTCCAATACGCATTCGTCCATC
12	*ISL1*	24-33	GTACGGGATCAAATGCGCCAAG	AGGCCACACAGCGGAAACACTC
13	*MALL(BENE)*	21-33	CATTACATCCGTGGATTCTCC	GTGCTTAAGAGAATGTGAGGG
14	*PLAU*	21-31	CGCTGCTCCCACATTGGCTAAG	TGTGCATGGGTGAAGGGAGAGC
15	*RAB25*	21-33	TGATCGGCGAATCAGGTGTGG	CAACATCACAGTGCGGGTGGAG
16	*S100A2*	22-27	GCAGCCTGGATGAGAACAGTGACC	CAGCCCTGGAAGAAGTCATTGCAC
17	*S100P*	18-32	GTCTGAATCTAGCACCATGACG	GGAAGCCTGGTAGCTCCTTC
18	*SLCO4A1*	22-32	TCCATCTGGCTCCTGCTGAAGAAC	GCTTCTGAGGCACTCAGGCTGAAC
19	*STMN1*	21-25	AAAGAACTGGAGAAGCGTGCCTCAG	CTGAATTTCCTCCAGGGAAAGATCC
20	*TGM2*	21-36	CTGGTCACTAACCAACAAGGTTG	GAGCAGGAGATAAAGTCAAAGCTG

Ct: cycle threshold.

### Gene pathways, networks, and ontology analysis

Biological networks were generated with Ingenuity Pathways Analysis 7.0 (IPA), a web-based application http://www.Ingenuity.com that enables the visualization and analysis of biologically relevant networks to enable the discovery, visualization, and exploration of therapeutically relevant networks as described previously [33]. Ontology analysis was performed with IPA and http://geneontology.org/.

### Immunohistochemistry

Routine immunohistochemistry was performed using formalin-fixed, paraffin-embedded sections as described in the manufacturer's protocol. We could obtain only RAB25 and S100P antibodies. The following antibodies, dilutions, and pretreatment conditions were used: anti-RAB25 (1:100), trypsin pretreatment; Abnova Corporation, Taipei, Taiwan) and anti-S100P (polyclonal rabbit anti-S100, (1:400), trypsin pretreatment; DakoCytomation, Copenhagen, Denmark).

### Statistical analysis

For statistical analysis program, we performed a logarithmic (log_2_) transformation of the data to stabilize the variance and the gene expression profile of each cancer cell line, which was normalized to the median gene expression level for the entire sample set. DNA microarray results of the eight test cell lines were analyzed with a Dunnett's test. qPCR results of the 19 candidate genes in 12 cell lines were analyzed with a Dunnett's test and principal component analysis (PCA). PCA was performed using both the PCA program in R (2.8.0; http://www.r-project.org/) and Microsoft Office Excel 2003 (Microsoft, Redmond, WA, USA).

## Competing interests

The authors declare that they have no competing interests.

## Authors' contributions

TW: Experimental design, all experiments, interpretation of data, and manuscript preparation. TM: Experiments (cell culture and DNA microarray analysis). YD: Experiments (cell culture and quantitative real-time PCR analysis). YF: Experiments (cell culture and quantitative real-time PCR analysis). MI: Experiments (analysis with antibody). MK: Experiments (analysis with antibody). CF: Experimental design, conception, and manuscript preparation. All authors have read and approved the final manuscript.

## Supplementary Material

Additional file 1 **One hundred genes examined in the present study.** Symbols, gene names and accession numbers are shown. Click here for file
