# Supplementary material for: Comparison of lung cancer cell lines representing four histopathological subtypes with gene expression profiling using quantitative real-time PCR
Source: Cancer Cell Int. 2010 Jan 21;10:2. doi: 10.1186/1475-2867-10-2 (PMC2817686; doi:10.1186/1475-2867-10-2)
Supplement: Additional file 1 — One hundred genes examined in the present study. Symbols, gene names and accession numbers are shown. [file 1475-2867-10-2-S1.pdf]

# **Additional file 1 – One hundred genes examined in the present study**

| No. | Symbol | Gene name | Accession number |
|---|---|---|---|
| 1 | *ABLIM1* | actin binding LIM protein 1 | NM_001003407 |
| 2 | *ACTB* | actin, beta | NM_001101 |
| 3 | *AMY2A* | amylase, alpha 2A (pancreatic) | NM_000699 |
| 4 | *ANGPTL4* | angiopoietin-like 4 | NM_139314 |
| 5 | *ANXA8* | annexin A8 | NM_001040084 |
| 6 | *APLP1* | amyloid beta (A4) precursor-like protein 1 | NM_001024807 |
| 7 | *AREG* | amphiregulin | NM_001657 |
| 8 | *ARF4L* | ADP-ribosylation factor-like 4D | NM_001661 |
| 9 | *ASCL1* | achaete-scute complex homolog 1 (Drosophila) | NM_004316 |
| 10 | *ATM* | ataxia telangiectasia mutated | NM_000051 |
| 11 | *ATP11A* | ATPase, class VI, type 11A | NM_015205 |
| 12 | *ATP6V1F* | ATPase, H+ transporting, lysosomal 14kDa, V1 subunit F | NM_004231 |
| 13 | *BEX1* | brain expressed, X-linked 1 | NM_018476 |
| 14 | *BICD2* | bicaudal D homolog 2 (Drosophila) | NM_001003800 |
| 15 | *BTBD3* | BTB (POZ) domain containing 3 | NM_014962 |
| 16 | *BTF3* | basic transcription factor 3 | NM_001037637 |
| 17 | *CANX* | calnexin | NM_001746 |
| 18 | *CD24* | CD24 molecule | NM_013230 |
| 19 | *CDC7* | cell division cycle 7 homolog (S. cerevisiae) | NM_003503 |
| 20 | *CDH1* | cadherin 1, type 1, E-cadherin (epithelial) | NM_004360 |
| 21 | *CFL1* | cofilin 1 (non-muscle) | NM_005507 |
| 22 | *CLDN4* | claudin 4 | NM_001305 |
| 23 | *COL7A1* | collagen, type VII, alpha 1 | NM_000094 |
| 24 | *CSTA* | cystatin A (stefin A) | NM_005213 |
| 25 | *CTSL1(CTSL)* | cathepsin L1 | NM_001912 |
| 26 | *CYB5A(CYB5)* | cytochrome b5 type A (microsomal) | NM_148923 |
| 27 | *DCK* | deoxycytidine kinase | NM_000788 |
| 28 | *DDB1* | damage-specific DNA binding protein 1, 127kDa | NM_001923 |
| 29 | *DST* | dystonin | NM_183380 |
| 30 | *DUSP4* | dual specificity phosphatase 4 | NM_001394 |
| 31 | *ECM1* | extracellular matrix protein 1 | NM_004425 |
| 32 | *EEF1B2* | eukaryotic translation elongation factor 1 beta 2 | NM_001959 |
| 33 | *EEF2* | eukaryotic translation elongation factor 2 | NM_001961 |
| 34 | *EFNA1* | ephrin-A1 | NM_004428 |
| 35 | *ELF3* | E74-like factor 3 | NM_004433 |
| 36 | *FABP1* | fatty acid binding protein 1, liver | NM_001443 |
| 37 | *FOSL1* | FOS-like antigen 1 | NM_005438 |
| 38 | *FOXG1* | forkhead box G1 | NM_005249 |
| 39 | *GAPDH(GAPD)* | glyceraldehyde-3-phosphate dehydrogenase | NM_002046 |
| 40 | *GNB1* | guanine nucleotide binding protein (G protein), beta polypeptide 1 | NM_002074 |
| 41 | *GPR116* | G protein-coupled receptor 116 | NM_015234 |
| 42 | *HBEGF* | heparin-binding EGF-like growth factor | NM_001945 |
| 43 | *HMGA1* | high mobility group AT-hook 1 | NM_145899 |
| 44 | *HPN* | hepsin (transmembrane protease, serine 1) | NM_002151 |
| 45 | *IFNGR2* | interferon gamma receptor 2 (interferon gamma transducer 1) | NM_005534 |
| 46 | *IGSF3* | immunoglobulin superfamily, member 3 | NM_001542 |
| 47 | *INA* | internexin neuronal intermediate filament protein, alpha | NM_032727 |
| 48 | *INADL* | InaD-like (Drosophila) | NM_176877 |
| 49 | *INSM1* | insulinoma-associated 1 | NM_002196 |
| 50 | *ISL1* | ISL LIM homeobox 1 | NM_002202 |
| 51 | *JUP* | junction plakoglobin | NM_021991 |
| 52 | *KIF5C* | kinesin family member 5C | NM_004522 |
| 53 | *KRT5* | keratin 5 | NM_000424 |
| 54 | *KRT13* | keratin 13 | NM_002274 |

| | | | |
|---|---|---|---|
| 55 | *KRT15* | keratin 15 | NM_002275 |
| 56 | *KRT17* | keratin 17 | NM_000422 |
| 57 | *KRTCAP3* | keratinocyte associated protein 3 | NM_173853 |
| 58 | *LDHA* | lactate dehydrogenase A | NM_005566 |
| 59 | *LGALS7* | lectin, galactoside-binding, soluble, 7 | NM_002307 |
| 60 | *MAL2* | mal, T-cell differentiation protein 2 | NM_052886 |
| 61 | *MALL(BENE)* | mal, T-cell differentiation protein-like | NM_005434 |
| 62 | *MCM3* | minichromosome maintenance complex component 3 | NM_002388 |
| 63 | *MGAT1* | mannosyl (alpha-1,3-)-glycoprotein beta-1,2-N-acetylglucosaminyltransferase | NM_002406 |
| 64 | *MYCL1* | v-myc myelocytomatosis viral oncogene homolog 1, lung carcinoma derived (avian) | NM_001033081 |
| 65 | *OCLN* | occludin | NM_002538 |
| 66 | *ODC1* | ornithine decarboxylase 1 | NM_002539 |
| 67 | *PDRG1* | p53 and DNA damage regulated 1 | NM_030815 |
| 68 | *PFDN5* | prefoldin subunit 5 | NM_002624 |
| 69 | *PHLDA1* | pleckstrin homology-like domain, family A, member 1 | NM_007350 |
| 70 | *PLAT* | plasminogen activator, tissue | NM_000930 |
| 71 | *PLAU* | plasminogen activator, urokinase | NM_002658 |
| 72 | *PLTP* | phospholipid transfer protein | NM_006227 |
| 73 | *PTMA* | prothymosin, alpha | NM_001099285 |
| 74 | *PTPRN2* | protein tyrosine phosphatase, receptor type, N polypeptide 2 | NM_002847 |
| 75 | *QPCT* | glutaminyl-peptide cyclotransferase | NM_012413 |
| 76 | *RAB25* | RAB25, member RAS oncogene family | NM_020387 |
| 77 | *RAB38* | RAB38, member RAS oncogene family | NM_022337 |
| 78 | *S100A2* | S100 calcium binding protein A2 | NM_005978 |
| 79 | *S100P* | S100 calcium binding protein P | NM_005980 |
| 80 | *SELENBP1* | selenium binding protein 1 | NM_003944 |
| 81 | *SFTPA1* | surfactant, pulmonary-associated protein A1 | NM_001093770 |
| 82 | *SFTPB* | surfactant, pulmonary-associated protein B | NM_000542 |
| 83 | *SFTPD* | surfactant, pulmonary-associated protein D | NM_003019 |
| 84 | *SLC16A4* | solute carrier family 16, member 4 (monocarboxylic acid transporter 5) | NM_004696 |
| 85 | *SLC25A6* | solute carrier family 25 (mitochondrial carrier; adenine nucleotide translocator), member 6 | NM_001636 |
| 86 | *SLCO4A1* | solute carrier organic anion transporter family, member 4A1 | NM_016354 |
| 87 | *SNAI2* | snail homolog 2 (Drosophila) | NM_003068 |
| 88 | *SPINT2* | serine peptidase inhibitor, Kunitz type, 2 | NM_021102 |
| 89 | *SSR2* | signal sequence receptor, beta (translocon-associated protein beta) | NM_003145 |
| 90 | *STMN1* | stathmin 1/oncoprotein 18 | NM_203399 |
| 91 | *TACSTD1* | tumor-associated calcium signal transducer 1 | NM_002354 |
| 92 | *TBL1XR1* | transducin (beta)-like 1 X-linked receptor 1 | NM_024665 |
| 93 | *TERT* | telomerase reverse transcriptase | NM_198253 |
| 94 | *TFRC* | transferrin receptor (p90, CD71) | NM_003234 |
| 95 | *TGM2* | transglutaminase 2 (C polypeptide, protein-glutamine-gamma-glutamyltransferase) | NM_004613 |
| 96 | *TMED9* | transmembrane emp24 protein transport domain containing 9 | NM_017510 |
| 97 | *TMSL8(TMSNB)* | thymosin-like 8 | NM_021992 |
| 98 | *TP63(TP73L)* | tumor protein p63 | NM_003722 |
| 99 | *UNG* | uracil-DNA glycosylase | NM_003362 |
| 100 | *VEGFC* | vascular endothelial growth factor C | NM_005429 |
